# Surgical explantation of failed transcatheter heart valves: indications and results

**DOI:** 10.1007/s00380-022-02119-7

**Published:** 2022-07-08

**Authors:** Andrea Muensterer, Nazan Puluca, Hendrik Ruge, Keti Vitanova, Ruediger Lange

**Affiliations:** 1 Department of Cardiovascular Surgery, German Heart Center Munich, Technical University Munich, Lazarettstr. 36, 80636 Munich, Germany; 2 Insure (Institute for Translational Cardiac Surgery), Department of Cardiovascular Surgery, German Heart Center, Technical University Munich, Munich, Germany; 3 DZHK (German Center for Cardiovascular Research), Partner Site Munich Heart Alliance, Munich, Germany

**Keywords:** Transcatheter aortic valve implantation, TAVR, THV, Surgical explantation

## Abstract

Given the recent surge in transcatheter heart valve replacement (THVR), cardiac surgeons will surely face the challenge of eventual explantation. The aim of this study was to determine indications for reoperation, while exploring pertinent technical aspects and survival after THV explantation in a cohort originally deemed high risk or even inoperable. Between February 2008 and March 2019, 31 patients with failed transcatheter aortic valve replacement (TAVR) underwent surgical explantations at our facility. Data were prospectively collected for retrospective analysis of procedural indications, technical issues, and postoperative survival. The major reason for TAVR removal was bioprosthetic valve failure (BVF) due to infective endocarditis (IE: 16/31 [51.6%]), non-structural (NSVD: 14/31 [45.2%]) and structural (SVD: 1/31 [3.2%]) valve deterioration accounting for the rest. Mean age at THV explantation was 76.3 ± 8.3 years, and median time from TAVR to explantation was 153 days (0 days–56.6 months). Median ICU and hospital stay were 6 days (1–44 days) and 23 days (8–62 days), respectively. Thirty-day and 1-year survival rates were 74.2% and 67.2%, respectively. Median follow-up interval after explantation was 364 days (3 days–80 months). Mean cardiopulmonary bypass time was 124.6 ± 46.8 min, and mean aortic cross-clamp time was 84.3 ± 32.9 min. There was no need for unplanned aortic root repair owing to tissue damage during dissection of the TAVR from surrounding tissue. The most common reason for THV explantation was (a) BVF for IE and (b) BVF secondary to NSVD. Although 30-day and 1-year mortality rates in this multimorbid cohort were predictably high, no procedural mortalities occurred.

## Introduction

Transcatheter aortic valve replacement (TAVR) is now a well-established procedure, no longer reserved for high-risk candidates only. In patients of intermediate risk, it is considered equivalent or superior to surgical aortic valve replacement (SAVR) [1, 2]. Therefore, increasing utilization of TAVR in intermediate and even low-risk patients can be expected. This may also present cardiothoracic surgeons with growing numbers of ensuing surgical explantations. Although data on feasibility, safety, and clinical outcomes of TAVR are rapidly accruing, less is known about outcomes of surgical THV explantation and subsequent SAVR.

Our intent was to determine indications for THV explantation, looking also at technical aspects of such procedures and survival rates in a cohort otherwise considered high risk or even inoperable.

## Patients and methods

### Study design and patient population

All patients undergoing surgical explantation of a THV at our center were identified via an institutional database. The intent was to gather information on THV explantation as a stand-alone procedure. All instances of emergency intraoperative conversion from TAVR to SAVR were summarily excluded. Data were collected prospectively and analyzed in retrospect, reporting mortality according to Valve Academic Research Consortium (VARC)-2 criteria [3]. Indications for THV removal were categorized using standard definitions of structural deterioration and valve failure in assessing long-term durability of transcatheter and surgical aortic bioprosthetic valves [4].

### Study endpoints

The primary study endpoint was to identify chief indications for THV removal. Secondary study endpoints were early and mid-term survival and analysis of specific surgical considerations for THV explantation.

### Surgical technique

TAVR explantation procedures were performed through median or partial upper sternotomy and under routine cardiopulmonary bypass (CPB) support. Exposure of the THV was achieved through standard aortotomy.

### Statistical analysis

Categorical variables were expressed as percentages, reporting continuous variables as mean (± SD) or median (range) values. Thirty-day and 1-year survival rates were plotted by Kaplan–Meier method. All analytics were driven by customary software (SPSS v25/IBM Corp/Armonk/NY/USA).

## Results

### Baseline patient characteristics

**Fig. 1:**
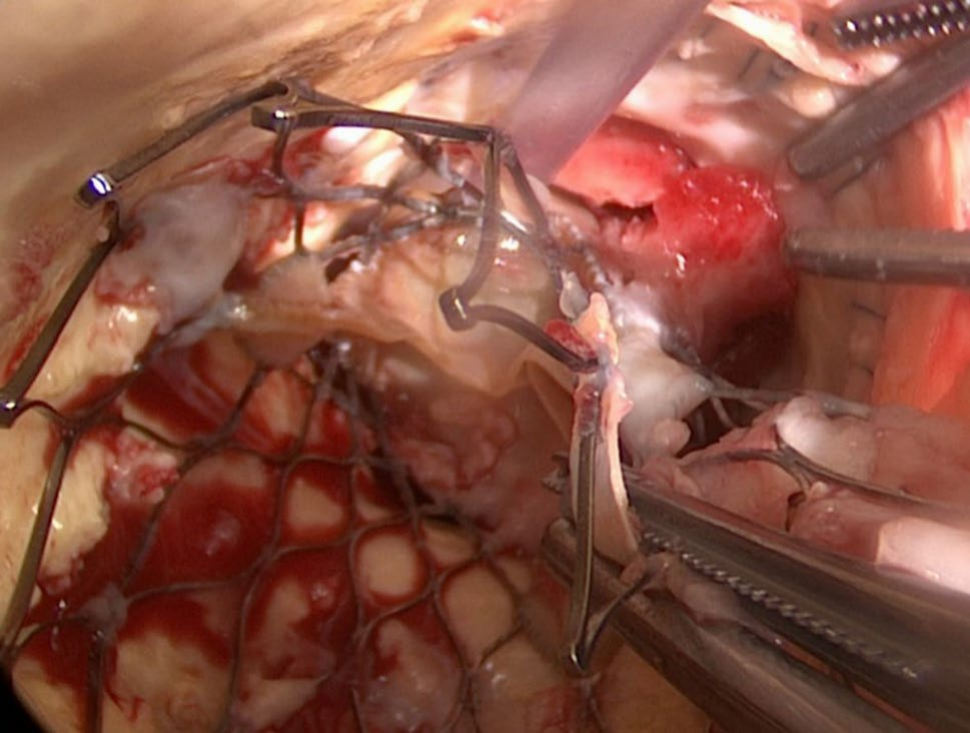
Explantation of a CoreValve prosthesis due to infective endocarditis

Between February 2008 and March 2019, a total of 31 patients submitted to surgical explantation of failing THV. During the same time period, 2568 TAVR procedures were performed in our centre. There were 23 men (74.2%), and the mean age was 76.3 ± 8.3 years. Median time between TAVR and valve explantation was 153 days (0 days–56.6 months). Initial TAVR in 16 patients (51.6%) were performed at our institution. Prior to TAVR, the mean Society of Thoracic Surgeons (STS) score was 3.0 ± 1.2%, and the mean logistic European System for Cardiac Operative Risk Evaluation (EuroSCORE) ranking was 12.6 ± 9.3%. Before TAVR explants, the corresponding values were 5.9 ± 5.0% and 25.1 ± 16.8%. Five patients (16.1%) had histories of prior conventional cardiac surgery, including aortic valve surgery. Baseline procedural patient characteristics are shown in Table 1, with more detailed profiling of patients in Table 2. Explantation of a CoreValve prosthesis (Medtronic/Dublin/Ireland) in a patient with IE (Infective Endocarditis) is depicted in Fig. 1.

**Table 1:** Baseline procedural patient characteristics

Sex, *n* (%)	
Male	23 (74.2%)
Female	8 (26.8%)
Age, years (mean ± SD)	76.3 ± 8.3
Previous cardiac surgery, *n* (%)	5 (16.1%)
Risk prior to TAVR,% (mean + SD)	
STS score	3.0 ± 1.2
Logistic EuroSCORE	12.6 ± 9.3
Risk at TAVR explantation,% (mean ± SD)	
STS score	5.9 ± 5.0
Logistic EuroSCORE	25.1 ± 16.8
Indication for explantation, *n* (%)	
IE	16 (51.6%)
NSVD	14 (32.3%)
Severe hemodynamic SVD	1 (3.9%)
Time from TVR to explantation, median (range)	153 days (0 days–56.6 months)
Follow-up after AVR, median (range)	364 days (3 days–80 months)

*SD* standard deviation, *TAVR* transcatheter aortic valve replacement, *IE* infective endocarditis, *NSVD* non-structural valve deterioration, *SVD* structural valve deterioration, *AVR* aortic valve replacement

**Table 2:** Comprehensive patient profiles

Patient number	Age at time of TAVR explantation (years)	Sex	TAVR device	logEuroSCORE at time of AVR procedure	STS score at time of AVR procedure	Previous cardiac surgery (yes/no, CABG/valve)	LVEF (%)	Indication for explantation	Time between TAVR and AVR (days)	CPB time (min)	Cross-clamp time (min)	Concomitant procedure	Hospital stay (days)	ICU stay (days)	Follow-up after AVR (days)	Status
1	83	Male	SEV	10,32	7,4	No	50	NSVD	11	96	56	None	21	5	2060	Alive
2	80	Male	SEV	6	2,5	No	55	NSVD	7	108	84	None	25	4	472	Dead
3	75	Female	SEV	9	21	No	60	NSVD	1	89	60	None	8	3	2	Dead
4	82	Male	SEV	26,7	4,6	No	33	NSVD	8	100	70	MVR	44	44	25	Dead
5	71	Female	BEV	25,54	4,25	Yes/valve (AVR homograft)	50	IE	186	106	78	None	14	9	2433	Alive
6	80	Female	SEV	20,5	3,5	No	30	NSVD	8	73	50	None	23	12	2204	Alive
7	65	Male	BEV	29,23	4,5	No	34	IE	462	153	71	CEA	16	12	255	Alive
8	72	Male	BEV	6,55	1,8	No	55	NSVD	27	224	94	CABG	21	2	2212	Alive
9	70	Male	BEV	10,2	1,6	No	45	NSVD	3	201	103	None	14	5	1152	Alive
10	76	Female	BEV	18,49	4,7	No	60	IE	147	174	143	MVR + CABG	16	1	1417	Alive
11	78	Female	BEV	28,38	7,68	No	60	NSVD	8	64	43	None	32	1	478	Alive
12	80	Male	SEV / BEV	44,66	3,42	Yes/valve (AVR, HAART-ring)	50	NSVD	61	107	65	None	10	2	502	Alive
13	79	Male	SEV	45,4	13,8	Yes/valve (AVR, CABG)	60	IE	579	138	100	None	62	43	1207	Alive
14	49	Female	SEV	3,13	0,9	No	40	severe hemodynamic SVD	1596	100	81	None	14	3	567	Alive
15	77	Male	SEV	17,47	3,88	Yes/valve (AVR, CABG)	55	NSVD	566	132	87	None	18	1	364	Alive
16	84	Male	SEV 2x	17,51	2,37	No	46	NSVD	1	76	49	None	42	35	43	Dead
17	75	Male	BEV 2x	26,7	3,2	Yes/valve (AVR Toronto Root)	61	IE	1722	178	130	CABG	25	5	667	Alive
18	75	Male	BEV	16,88	2,53	No	45	IE	83	87	63	None	35	5	897	Alive
19	77	Male	SEV	28,75	3,59	No	45	NSVD	449	228	154	MVR + TVR + replacement ascending aorta	23	14	14	Dead
20	78	Male	BEV	64,53	2,78	No	35	IE	233	125	95	None	20	6	662	Alive
21	86	Male	BEV	29,71	4,49	No	43	IE	876	107	81	CABG	31	31	30	Dead
22	75	Female	BEV	39,60	8,56	No	41	IE	153	152	109	MVR + TVR	21	7	245	Alive
23	81	Male	SEV	7,3	2,1	No	56	NSVD	0	158	86	None	11	5	395	Alive
24	82	Female	SEV	53,6	16,6	No	45	IE	169	67	44	None	23	23	19	Dead
25	83	Male	BEV	37,13	14,4	No	50	IE	757	54	36	None	29	22	22	Dead
26	79	Male	BEV	16,34	2,84	No	57	IE	1594	114	97	None	12	2	143	Alive
27	53	Male	SEV	26,8	14,4	No	45	IE	490	117	89	MVR	11	11	11	Dead
28	71	Male	BEV	4,92	1,566	No	45	NSVD	76	99	63	VSD-closure	62	7	161	Dead
29	82	Male	SEV	20,16	7,696	No	65	IE	328	110	66	none	50	29	49	Alive
30	84	Male	BEV	19,56	3,753	No	55	IE	408	209	179	MVR	25	4	75	Alive
31	83	male	BEV	68,79	6,404	No	50	IE	45	116	88	None	26	25	25	Dead

*TAVR* transcatheter aortic valve replacement, *STS score* Society of Thoracic Surgeon score, *CABG* coronary artery bypass graft, *LVEF* left ventricular ejection fraction, *AVR* aortic valve replacement, *CPB* cardiopulmonary bypass, *ICU* Intensive Care Unit, *NSVD* non-structural valve deterioration, *SVD* structural valve deterioration, *IE* infective endocarditis, *MVR* mitral valve replacement, *CEA* carotid endarterectomy, *TVR* tricuspid valve repair, *LVOT* left ventricular outflow tract, *PPM* prosthesis-patient-mismatch, *BEV* balloon-expandable valve, *SEV* self-expandable valve

### Indications for transcatheter heart valve explantation

The most common indication for explantation was bioprosthetic valve failure (BVF) secondary to IE (16/17) or severe hemodynamic structural valve deterioration (SVD) (1/17), all explants undertaken after a 408-day median (45–1722 days). Second in frequency was BVF secondary to non-structural valve deterioration (NSVD: 14/31 [45.2%]), including paravalvular leakage (PVL, 10/14), THV dislocation (2/14), prosthesis-patient mismatch (PPM, 1/14), and failed aortic reconstruction (1/14). In patients presenting this way, THV explantation was performed after a median of 8 days (0–566 days). PCR test results were available in 10 patients (negative 7/10, positive 3/10) with IE. There was a variety of causative organisms in patients with IE. Information was available in 15/16 cases. *Staphylococcus aureus* (3/15), *Staphylococcus epidermidis* (3/15), *Staphylococcus aureus* (2/15), *Staphylococcus capitis*/*Abiotrophia defectiva*/*Staphylococcus haemolyticus*/*Lactococcus garciae*/*Cardiobacter hominis*/*Streptococcus agalacticae/Streptococcus mitis *(1/15).

### Explanted prostheses

There were two patients with existing THV implants during THVR procedures. Consequently, 33 prostheses were explanted from 31 patients, including 18 self-expandable valves (SEVs: Medtronic CoreValve,12; Medtronic Evolut R, 2; Medtronic Engager, 1; Edwards Centera, 1; Symetis [Boston Scientific/Marlborough/MA/USA], 2) and 15 balloon-expandable valves (BEVs: Edwards SAPIEN XT,9; Edwards SAPIEN 3,6).

SEVs were explanted for BVF secondary to NSVD (*n* = 11), IE (*n* = 6), or SVD (*n* = 1). BEVs were explanted for BVF secondary to IE (*n* = 10) or NSVD (*n* = 5). One patient with both SEV and BEV implants was discounted when calculating respective operative times.

### Technical aspects of transcatheter heart valve explantation

In 24 of 31 patients, there were no substantial adhesions to hamper THV removal from native aortic annulus. For the remainder of patients (SEV, 1; BEV, 6), explantation was described as arduous, requiring dissection of dense adhesions to prevent collateral tissue damage. BVF was attributable to IE (*n* = 6) or NSVD (*n* = 1).

### Operative results and clinical outcomes

At explantation, patients underwent median re-sternotomy or partial sternotomy and AVR using either a bioprosthesis (*n* = 30) or a mechanical prosthesis (*n* = 1). Peri- and intraoperative data are summarized in Tables 3 and 4.

**Table 3:** Ancillary peri- and intraoperative data

Concomitant procedures, *n* (%)	8 (30.8%)
CABG	
MVR	3
MVR + CABG	1
MVR + TVR + ascending aorta	1
replacement	1
MVR + TVR	1
CEA	1
CPB time, mean ± SD, min	124.6 ± 46.8
Aortic cross-clamp time, mean ± SD, min	84.3 ± 32.9
Total operative time (mean ± SD), min	268.2 ± 89.3
ICU stay, mean (range)	6 (1–44)
Hospital stay, mean (range), days	23 (8–62)

*CABG* coronary artery bypass craft, *CPB* cardiopulmonary bypass, *ICU* Intensive Care Unit, MVR: mitral valve replacement, *CEA* carotid endarterectomy, *TVR* tricuspid valve repair

**Table 4:** Intraoperative THV explantation data

	CPB time (mean ± D)		Aortic cross-clamp time (mean ± SD)		Total operative time (mean ± SD)	
BVF secondary to:						
NSVD	125.4 ± 55.6 min	*p* value: 0.996	76 ± 29 min	*p* value: 0.196	276.4 ± 109.3 min	*p* value: 0.701
IE	125.44 ± 40.8 min		91.8 ± 36.3 min		263 ± 73.9 min	
Isolated SAVR: BVF secondary to:						
NSVD	110.4 ± 42.6 min	*p* value: 0.429	68.3 ± 20.3 min	*p* value: 0.706	259.6 ± 73.8 min	*p* value: 0.257
IE	97.7 ± 23.8 min		72.8 ± 21.6 min		224.3 ± 56.7 min	
Isolated SAVR						
BEV	115 ± 39.7 min	*p* value: 0.373	99.4 ± 32.5 min	*p* value: 0.101	261.9 ± 50.3 min	*p* value: 0.478
SEV	81.4 ± 19.9 min		65.0 ± 20.7 min		239.2 ± 85.2 min	

*THV* transcatheter heart valve, *CPB* cardiopulmonary bypass, *BVF* bioprosthetic valve failure, *NSVD* non-structural valve dysfunction, *IE* infective endocarditis, *SAVR* surgical aortic valve replacement, *BEV* balloon-expandable valve, *SEV* self-expandable valve, *SD* standard deviation

A total of 20 patients (NSVD, 10; IE, 9; SVD,1) underwent THV explantation (SEV, 13; BEV, 8) and subsequent SAVR as isolated cardiac procedures. In eleven patients (35.5%), concomitant procedures were performed as follows: coronary artery bypass graft (CABG), 3; carotid endarterectomy (CEA), 1; mitral valve replacement (MVR), 3; MVR + CABG,1; MVR + tricuspid valve repair (TVR), 1; MVR + TVR and replacement of ascending aorta, 1; and closure of ventricular septal defect (VSD), 1.

MVR was necessary due to mitral valve IE, 3 and concomitant mitral valve insufficiency, 3.

Median ICU stay was 5 days (1–44 days) in patients with NSVD and 10 days (1–43 days) in patients with IE (*p* = 0.150). Median hospital stay was 10 days (8–62 days) for NSVD and 24 days (11–62 days) for BVF (*p* = 0.662).

**Fig. 2:**
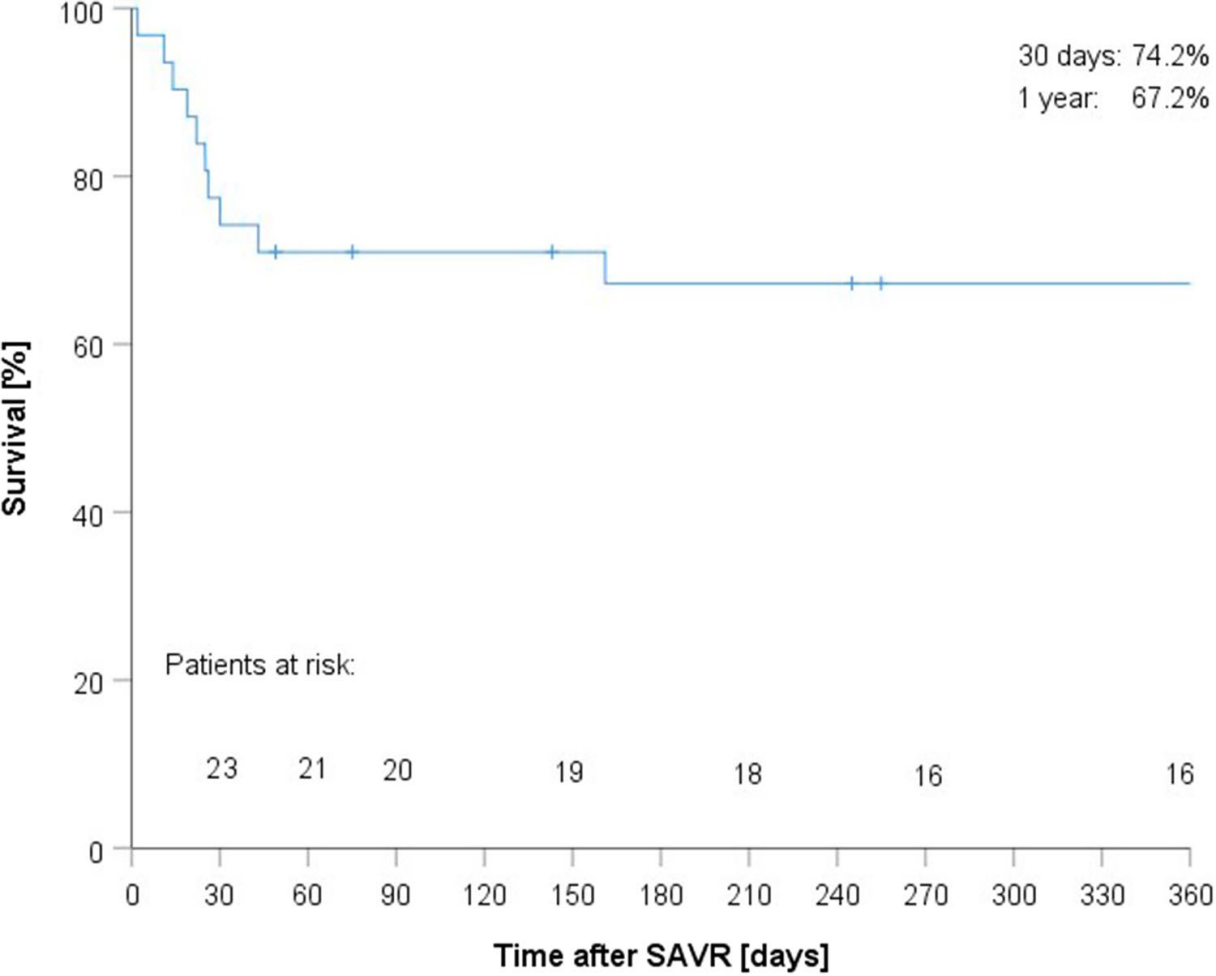
Kaplan–Meier plot of overall patient survival

Eleven patients died during follow-up (IE, 6; NSVD,5). Median follow-up time after THV explantation was 364 days (2–2433 days). Median follow-up time after THV explantation was 433 days (2–2212 days) for NSVD-related BVF and 194 days (11–2433 days) for IE-related BVF. Estimated 30-day and 1-year survival rates were 74.2% and 67.2%, respectively. As defined by VARC-2 criteria, all deaths were cardiovascular in nature. Figure 2 is a Kaplan–Meier plot of overall patient survival. The 30-day and 1-year survival estimates were the same (68%) in patients with IE, differing distinctly (80% and 66.7%, respectively) in those with other reasons for THV explantation.

## Discussion

In the present study, we examined early and mid-term outcomes of 31 patients undergoing surgical explantation of failed TAVR prostheses. Given the popularity of TAVR procedures, THV failure and the need for surgical explantation may soon become a pressing issue [5]. However, little is known regarding modes of THV failure, treatment entailed, and outcomes in instances where surgical explantation is required.

Mylotte et al. [6] have cited endocarditis of prosthetic valves, SVF, and valvular thrombosis as modes of failure tantamount to surgical bioprosthetic failure. Indeed, late THV embolization and chest compressions that damage THV implants during cardiopulmonary reanimation seem unique to THV as modes of failure, as do PVL and stent fractures. Still, only case reports of THV explantation for BVF or NSVD are chiefly available at present [7, 8]. Fukuhara et al. [9] studied 17 patients undergoing TAVR explantation on the basis of PVL (7/17), SVD (4/17), TAVR procedural complications (4/17), and IE (1/17) or as a bridge to definitive open surgery (1/17). They ultimately determined that risk scores (STS-Score) at time of TAVR procedures and those at times of explantations differed significantly (9.9% vs 3.5%; *p* < 0.001). Neoendothelization of THV implants prompted aortic root repairs in two cases.

### Infective endocarditis (IE)

The 5-year incidence of IE after TAVR is at 5% [10] and was the most common indication for THV explantation in our cohort. Other studies have indicated no difference between SAVR and TAVR in terms of IE risk [11, 12]. However, it is feasible that differing implantation techniques used for BEVs and SEV cause disparities in IE. Thus far, the limited data on this subject has produced conflicting results. Reguiero et al. were focused on post-TAVR IE in their analysis of 245 patients, finding no difference in early or late mortality or reoperation rate by prosthesis type (BEV vs SEV) [13]. On the other hand, Mylotte et al. [6] and Amat-Santos et al. have separately recorded higher rates of post-TAVR IE for BEV (vs SEV) implants (59% vs 41%, and 64% vs 36%, respectively), with the reverse being documented by Brouwer et al. (SEV, 56%; BEV, 44%) [14].

There are three studies reporting onset of IE at 6 months after TAVR [13–15], whereas Butt et al. seem to refute this, citing a median of 12 months for rehospitalization due to IE [10]. In our study, IE-related prosthetic failures were explanted 368 days after prior TAVR. This discrepancy between onset of IE and final surgical treatment suggests that patients admitted with post-TAVR IE are treated conservatively for prolonged periods [14, 15] before considering open explantation and referred late for surgical intervention. There are lacking data on ratio and outcome between surgical and medical management for THV endocarditis. Future studies or register analysis are needed to evaluate this matter.

### Paravalvular leakage (PVL)

Moderate or severe PVL after TAVR may significantly influence long-term outcomes [16, 17]. In a systematic review, the pooled estimate for overall incidence of moderate or severe PVL was 11.7% for first-generation THV devices [18]. During the PARTNER IB trial, moderate to severe PVL was associated with an increased 5-year mortality risk in the TAVR group [19]. Although PVL is potentially treatable through interventional therapies [20], PVL emerged in our series as the second most common indication for surgical THV explantation, with more SEV devices explanted due to PVL (SEV,8; BEV,2). This outcome is aligned with data published elsewhere showing a higher rate of PVL in first-generation SEVs [21]. PVL rates in new third-generation SEVs and BEVs do not show such differences [22].

Although post-TAVR PVL is still a concern, the risk may be lowered by accurate annular measurement, adequate valve sizing, and precise positioning of the valve. Second- and third-generation THV prostheses allow repositioning during implantation and are equipped with an external sealing skirt to reduce the incidence of PVL. There were 8/10 THV explantations due to PVL in the first 16 of our patients, and all involved first-generation devices. In the 15 patients that followed, only 2/10 required explantations were linked to PVL. This issue may then be dramatically lessened by current and upcoming device generations [17, 23].

### Hemodynamic structural valve deterioration (SVD)

Recent studies have validated the good long-term functional results of TAVR devices [24] in ~ 7 years of accrued durability data [25]. However, as TAVR procedures trend higher in younger patients, prosthetic degeneration may become problematic. Two case reports of TAVR in patients < 60 years old have implicated early bioprosthetic valve failure[8, 26], the first case being a 53-year-old man with severe hemodynamic SVD 3 years after TAVR. The second report describes a 48-year-old woman initially slated for AVR. TAVR was performed as a rescue therapy for acute heart failure requiring CPR. This patient presented 4.4 years later with bioprosthetic valve failure due to severe hemodynamic SVD. Her left ventricular function had significantly improved after TAVR, allowing uneventful explantation and SAVR using a mechanical prosthesis. Van Steenberghe et al. insist that TAVR is contraindicated in operable patients [26], but we consider it a viable option in select cases, serving as a bridge to surgery for unstable or critically ill patients.

### Dislocation

After an initial learning curve, a dislocated or malpositioned THV device is very rare nowadays, owing to delivery catheters with repositioning features [33]. Dislocation may occur retrograde into the left ventricle or antegrade into the aorta. If such complications arise, conversion to conventional AVR is one possibility [27, 28], or the displaced prosthesis may be anchored in the ascending or descending aorta. During the present study, two patients required THV explantations due to left ventricular prosthetic valvular dislocations (0–3 days after TAVR). In the first patient, proper positioning of a BEV was initially achieved. Two days later, signs of acute heart failure had developed, the dislocated prosthetic valve identified in left ventricular outflow tract (LVOT) by echocardiography. Surgery showed, it was set transversely, causing near-complete LVOT obstruction. In the second patient, a BEV dislocated during implantation, becoming lodged in the mitral valve chords and stuck in the LVOT.

### Survival

In the present study, the mean STS score upon device explantation was 5.9%, and the mean logistic EuroScore was 25.1%, both quite consistent with a high-risk cohort. The 30-day and 1-year survival rates were 74.2% and 67.2%, respectively (**Fig. ****2**). IE is a serious medical condition, with a mortality rate of ~ 30% [15, 29], and was the chief indication for THV explantation in our cohort; so a high mortality rate was not unexpected.

All deaths were categorized as cardiovascular in nature. Interestingly, survival after THV explantation, whether due to IE or other causes, did not differ significantly. Patients with IE showed a complex clinical picture of sepsis leading to multiorgan failure, neurologic impairments, and eventual death. Additional studies are needed to further assess these findings.

### Technical aspects

There is only limited information on technical aspects of THV explantation, stemming from a case series where Fukuhara et al. examined TAVR explants of 17 patients. SAVR was performed > 12 months after TAVR in five of these subjects, each displaying degrees of device neoendothelialization that required “intense aortic endarterectomy”. Unplanned aortic root repairs were needed in two patients as well. As stated by Fukuhara et al., the importance of proper patient selection was duly underscored, noting the clear potential for aortic repair during TAVR explants [9].

THV explantations may also necessitate aortotomy modifications. Standard aortotomy is feasible for low-frame TAVR devices, such as the Edwards SAPIEN, whereas the higher stent frame of a Medtronic CoreValve device demands a slightly higher aortotomy. In this setting, suitable aortic access may be determined by palpating upper portions of the THV frames.

To achieve prosthetic removal, we largely resorted to blunt dissection. However, the ingrowth and solid adhesions of BEVs render explantation more difficult, compared with surgical bioprostheses. In the beginning, we used ice-cold saline to support mobilization of the nitinol frames. This proved unhelpful and was soon abandoned. Use of wire cutters to snip stent frames was beneficial for dislocated THVs caught up in left ventricle and mitral valve.

It is possible that BEVs may be more daunting than SEVs during explantations, given the high radial forces applied to such prosthetic valves and native tissues during implantations. The time intervals between TAVR and SAVR may also influence degree of neoendothelialization, further challenging THV dissections.

We did not incur any aortic wall or aortic root injury during operations, even if severe periprosthetic adhesions called for time-consuming dissection. In one case, however, patch reconstruction of the aortic annulus was necessary to rectify annular abscess formation in a patient with severe IE.

### Limitations

This study was confined to our single-center experience, hampered by a small sample size and a retrospective study design. None of the data generated has been reviewed by an independent adjudication committee.

## Conclusion

We found that the most common indication for THV explantation was IE. One-year survival in this high-risk patient population (considered ineligible for conventional surgery) was nearly 70%. There were more annular adhesions in conjunction with BEV (vs SEV) implantation, but no substantial damage to native aortic annulus or aortic root resulted from THVR.
